# A Novel Insight into the Immune-Related Interaction of Inflammatory Cytokines in Benign Prostatic Hyperplasia

**DOI:** 10.3390/jcm12051821

**Published:** 2023-02-24

**Authors:** Xiaokaiti Naiyila, Jinze Li, Yin Huang, Bo Chen, Mengli Zhu, Jin Li, Zeyu Chen, Lu Yang, Jianzhong Ai, Qiang Wei, Liangren Liu, Dehong Cao

**Affiliations:** 1 Department of Urology, Institute of Urology, West China Hospital, Sichuan University, Chengdu 610041, China; 2 West China School of Medicine, Sichuan University, Chengdu 610041, China; 3 Research Core Facility, West China Hospital, Sichuan University, Chengdu 610041, China

**Keywords:** benign prostatic hyperplasia, inflammation, microenvironment, inflammatory factor, aging

## Abstract

Benign prostatic hyperplasia (BPH) is a common male condition that impacts many men’s quality of life by generating lower urinary tract symptoms (LUTS). In recent years, inflammation has become very common in the prostate, and BPH with inflammation has a higher International Prostate Symptom Score (IPSS) score and an enlarged prostate. Chronic inflammation leads to tissue damage and the release of pro-inflammatory cytokines, which play an important role in the pathogenesis of BPH. We will focus on current advancements in pro-inflammatory cytokines in BPH, as well as the future of pro-inflammatory cytokine research.

## 1. Introduction

Benign prostatic hyperplasia (BPH), which is the most common chronic urinary disease in elderly men, is usually seen in men over 50 years old [1]. Much evidence suggests that modifiable factors in addition to aging, such as diet, obesity, hypertension, dyslipidemia, hormonal imbalances, alcohol, and smoking, can contribute to the development of BPH [2,3]. The main pathological manifestation of BPH is the overgrowth and remodelling of epithelial and fibromuscular tissue in the transitional zone of the prostate and around the urethra, resulting in enlargement of the prostate [4]. The most crucial risk factor for the occurrence and development of BPH is age, and the growth factor pathway is affected in elderly men, leading to tissue remodelling [5]. Blocking its associated growth factors or stimuli may be an effective way to treat BPH. However, there are still other factors contributing to the development of BPH, such as insulin, metabolic syndrome, proliferation and reawakening, stem cells, and telomerase pathways, in addition to the above two mechanisms [6,7]. Among them, the metabolic syndrome (MetS) and stem cell hypothesis is thought to be related to the inflammatory state of the prostate. It has been shown that patients with metabolic syndrome are at higher risk of developing BPH [8]. MetS is characterized by a systemic inflammatory state and is associated with elevated levels of IL-8, IL-6, IL-1β, TNF-α, and C-reactive protein [9,10]. A high-fat diet increases pro-inflammatory cytokines via the Signal Transducer and Activator of Transcription (STAT)-3 and Nuclear Factor-kappa B (NF-kappaB) pathways [11,12]. In terms of stem cells, a recent hypothesis proposes that when the prostatic transitional zone is exposed to antigens or urinary components, an inflammatory microenvironment forms, causing stem cells to differentiate into smooth muscle cells in response to inflammatory growth factors and cytokines, thereby replacing the natural smooth muscle cells around the periurethral area. Furthermore, it suppresses epithelial stem cells in the prostate’s basal niche in the transition zone, resulting in neoplastic benign growth and leading to nodular BPH [13]. We begin by looking at the androgen pathway, with a particular focus on the inflammatory pathway’s role in the development of BPH.

Androgens have long been thought to be involved in prostate growth. It is now clearer how the androgen system and androgen receptors contribute to BPH. Prostate tissue that has undergone hyperplasia expresses androgen receptors, which are then activated by the potent androgen dihydrotestosterone (DHT). Leimgruber et al. [14] demonstrated in vitro that testosterone action increased the proliferative capacity of rat prostate smooth muscle cells (PSMCs), as well as the expression of the proliferation-associated protein extracellular signal-regulated kinase 1/2 (p-ERK1/2). This suggests that androgens play a role in prostate tissue proliferation. Reducing androgen levels may prevent further prostate enlargement, resulting in a reduction in prostate volume. However, in recent years, the potentially important role of chronic inflammation in the pathogenesis of BPH has gradually emerged and is considered to be a key part in the occurrence and development of BPH. Men with acute or chronic inflammation had a higher risk of prostate volume and acute urinary retention (AUR) caused by BPH than men without acute or chronic inflammation [15,16]. In another prospective study of 167 male autopsy samples, 93 were found to be BPH patients, in which 75% of the examined prostates were found to have chronic inflammation, while only 50% of the glands without BPH had chronic inflammation [17].

BPH has a high frequency and incidence, and it has a significant influence on daily living. The cause of the disease is currently unknown. Despite the prevalence of a number of these theories, there is now mounting evidence that inflammatory variables play a substantial role in BPH [15,17]. As research improves, additional relationships between inflammatory factors and BPH have been reported. Consequently, we will discuss the role and relationship of pro-inflammatory cytokines in chronic inflammation, which is a critical component of BPH pathogenesis.

## 2. Androgen Pathway and Inflammation

Androgens, especially testosterone, play an essential role in the development and growth of the normal prostate. Most of the testosterone in the body comes from the testis (95%) and a small part comes from the adrenal gland (5%), which is the main serum androgen that stimulates the growth of the prostate [18]. During puberty, the hypothalamic pituitary gonadal axis is activated, the testis begins to synthesize a large amount of T, the prostate develops rapidly, and the volume and weight begin to increase [19]. After entering adulthood, the growth of the prostate enters a plateau and slows down, but in some middle-aged and elderly men, there may be a gradual increase in the volume of the prostate, that is, hyperplasia.

Many studies have confirmed the correlation of androgen in the occurrence and development of BPH. Sasagawa et al. [20] followed 13 patients with hypogonadism (ages 25–32). The prostate volume of patients with hypogonadism increased significantly after supplementation with exogenous testosterone. Before that, their prostate volume was significantly smaller than that of healthy men of the same age [21]. Although testosterone is the main androgen component in serum, it has been found that serum-circulating testosterone levels usually decrease with age [22]. Paradoxically, patients with hypogonadism who received androgen therapy had no increased risk of developing BPH [23]. 

In androgen-dependent tissues, such as the skeletal muscle, brain, and seminiferous epithelium, the androgen-dependent process is primarily influenced by androgens in the form of testosterone [4]. However, in prostate tissue, testosterone is catalyzed and converted into more effective dihydrotestosterone (DHT) by 5-hydroxytryptamine reductase (5-α-R) synthesized by prostate stromal cells, which plays a vital role in the formation and function of organs [19]. DHT acts mainly by binding to androgen receptor (AR). AR is widely expressed in benign epithelium and adjacent stroma. The affinity between DHT and AR is three times that of testosterone. Compared with normal tissues, the level of DHT in prostate tissue is higher but remains stable during aging [23]. The study demonstrated that in both healthy adults and elderly patients with BPH, the level of DHT in prostate tissue was approximately 10–20 times that of testosterone, while the level of DHT in serum was the opposite [24,25]. From the Pless study, 1524 patients with BPH received 5 milligrams of finasteride, a 5 α reductase inhibitor (5α RI), daily, and 1516 patients with BPH received a placebo. Four years later, the prostate volume in the finasteride group decreased by 18%, while that in the placebo group increased by 14% [26]. Roehrborn et al. [27] randomly divided 4325 patients with BPH into two groups: the dutasteride group and the control group. Following two years of therapy, serum DHT levels increased by 9.6% in patients taking placebo, while serum DHT levels decreased by 90.2% in patients taking dutasteride. The volume of the transitional zone of the prostate decreased by 25.7% in the dutasteride group and 12.4% in the control group. Clinically, the use of 5α RI has long shown its importance. In patients with BPH, the International Prostate Symptom Score (IPSS), maximum urinary flow rate, and reduction in prostate volume can be shown after five RI treatments [28]. Although 5α RI has effectively reduced the risk of progression of BPH in clinical practice, 10% of patients still have clinical progress [27,29]. This suggests that androgen may not be the only cause of BPH.

In the prostate, growth factors are released by stromal cells and maintain the dynamic balance of prostate cells by autocrine and paracrine signaling [30]. In prostatic hyperplasia fibroblasts expressing AR, fibroblast growth factor-2 (FGF-2) and fibroblast growth factor-7 (FGF-7) were overexpressed [31]. Transforming growth factor-β1 (TGF-β1) induces mesenchymal fibroblasts to differentiate into myofibroblasts and regulates the response of epithelial cells to the IGF-1-mediated mesenchymal–epithelial cell axis [32], leading to hyperplasia associated with BPH. The increase in the growth factor level is related to the occurrence of BPH. A recent study showed that pro-inflammatory macrophages induce an increase in BPH tissue interstitial hyperplasia through the AR signaling pathway. Using an in vitro co-culture system of human macrophages with different mesenchymal cells of the prostate, researchers performed immunohistochemical analysis with anti-CD68 and anti-AR antibodies and found that the number of macrophages and AR staining density were significantly higher in the mesenchyme of the transitional zone than in the peripheral zone [33]; AR expression was investigated by qPCR and AR mRNA expression was found to be significantly higher in the transitional zone compared with the peripheral zone; and in ARE-driven luciferase assay tested, AR-mediated ARE-type luciferase activity was found to be significantly higher in the transitional zone than in the peripheral zone [33]. All these experimental results suggest that infiltrating macrophages promote the proliferation of prostate mesenchymal cells through a mesenchymal AR-dependent pathway and that mesenchymal responses to macrophages differ between the migratory and peripheral bands due to differences in mesenchymal AR signaling. In addition, interstitial AR was also mentioned to be involved in the inflammatory process in a previous study, and the stromal AR/CCL3/stromal cell expansion signaling pathway was found, suggesting that the inflammatory signaling pathway may synergize with the AR signaling pathway to promote prostate stromal cell proliferation [34], which makes us focus on inflammation.

However, some studies have found that AR expression is lower in BPH primary epithelial cells (PEC) compared to normal PEC [1]. Reduced AR expression in BPH luminal cells is associated with increased regional prostate inflammation. Moreover, the causal relationship between the two has yet to be established conclusively [2]. Interfering with the AR signaling pathway in mouse intraluminal cells can promote local production of chemokines and cytokines, promoting AR-independent prostate epithelial cell proliferation [2]. The disparity between in vivo and in vitro experimental results suggests that the intrinsic link between AR expression and prostate inflammation is unknown, and deeper mechanisms need to be further explored [35,36].

## 3. Oxidative Stress and Inflammation

Through a number of pathways, chronic or acute inflammation can drive proliferative events and post-translational DNA alteration in prostate tissue, the most important of which is oxidative stress (OS) [37]. It is unknown what causes oxidative stress and oxidative DNA damage to cause accelerated prostate growth and other cellular alterations. OS is a cellular environment state. OS arises when there is an imbalance between the production of reactive oxygen clusters (ROS) and the biological system’s ability to repair oxidative damage or neutralize active intermediates (such as peroxides and free radicals) [38]. High levels of ROS generation can result in a major drop in antioxidant defense systems, resulting in protein, fat, and DNA damage, as well as consequent disruption of cell function and cell death, whereas low amounts can result in subtle changes in intracellular signaling pathways [39,40]. The deterioration of the antioxidant defense system will exacerbate oxidative damage [41].

Some researchers suggest that the emergence of OS may have two different effects [42]. To begin, oxidative stress can directly boost growth activity and stimulate signal pathways such as MAP kinase and PI3K/AKT, and therefore cause cell proliferation [43,44]. Second, oxidative damage to DNA can cause cell senescence in prostate epithelial cell subsets and the production of a number of cytokines via aging-related secretory responses [45]. The researchers discovered that the content of the oxidative stress marker 8-OH deoxyguanosine (8-OH-DG) in human BPH tissue was significantly higher than in normal transitional zone tissues from the control group, and that the level of 8-OHdG was correlated with prostate weight. ARR2PB-NOX4 (ARR2PB-NADPH oxidase 4) transgenic mice induced oxidative stress via Nox4, resulting in increased oxidative DNA damage in the prostate, increased prostate weight, increased epithelial cell proliferation, and histological changes including epithelial cell proliferation, interstitial thickening, and fibrosis [42]. As a result, their study demonstrated that oxidative stress and DNA oxidation play an essential role in the pathogenesis of BPH.

Furthermore, investigations have shown that in an animal model of BPH, lipid peroxidation of the prostate rose dramatically, whereas antioxidant enzyme activity such as glutathione peroxidase (GPX), catalase (CAT), and superoxide dismutase (SOD) declined [46]. As a result, monitoring lipid, protein, and DNA oxidation levels, as well as changes in antioxidant concentration, can be utilized as a diagnostic approach [38]. In this context, the use of antioxidants can increase the therapeutic efficacy of BPH [47]. At the moment, oxidative stress is one of the processes initiated by prostate gland inflammation which destroys the target tissue and impacts prostate cell growth. Consequently, inflammation and oxidative stress play a role in the pathophysiology of BPH.

## 4. Inflammation and Pro-Inflammatory Cytokines

In recent years, prostate tissue has been exposed to many irritants from the urinary tract over time, and there are many immune cells in normal prostate tissue, of which the proportion of T lymphocytes is more than 90%, with CD8+ T cells as the main component usually located in the periglandular region, while CD4+ T cells are distributed in the stroma [48]. Clinical evidence shows that chronic inflammation is not only the key condition leading to prostate enlargement and increased symptom scores but also the main risk factor for complications [1]. The longitudinal results of the prostatic symptom medical therapy study (MTOPS) provide clinical evidence that inflammation is important in BPH and LUTS. In baseline prostate biopsies, 43% of men had chronic inflammation. Di Silverio et al. [49] found that 69% of the inflammation belonged to chronic inflammation, and the inflammation in the prostate increased significantly with increasing prostate volume and age.

The origin of chronic inflammation may arise from several different stimuli, including bacterial infection (*E. coli*), viral infection (human herpes simplex virus, human papillomavirus, and cytomegalovirus), sexually transmitted organisms (*Treponema pallidum*, *Neisseria gonorrhoeae*, *Trichomonas vaginalis*, and chlamydia), dietary factors, autoimmune response, hormones, and urine reflux to the prostate collecting duct [50]. The persistence of one or more of these stimuli may lead to chronic inflammation. Against this background, Steiner et al. [51] found an increase in macrophages and lymphocytes (CD45) in inflammatory infiltration. Among them, 70–80% were CD-3T lymphocytes, and 10–15% were CD-19 and CD-20B lymphocytes. Interestingly, a reversal of the ratio of CD4 to CD8 T cells can be seen in samples of BPH tissues, where most T lymphocytes express CD4 [51,52].

**Figure 1:**
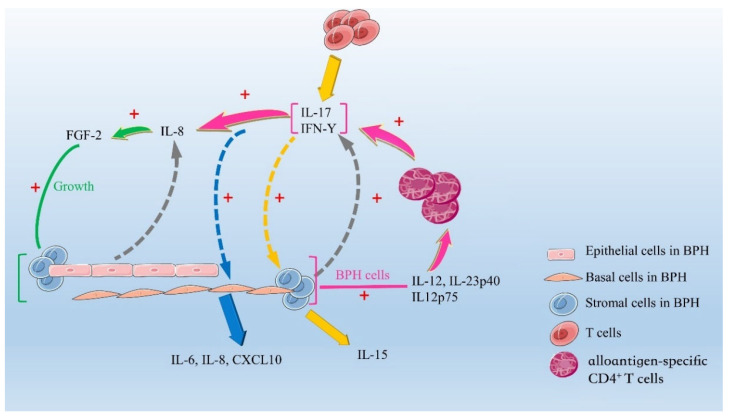
Pro-inflammatory microenvironment. The quantity of IL-8 generated by epithelial cells and IFN-γ produced by basal and stromal cells increases in a chronic inflammatory state (shown by black arrows). FGF-2 is upregulated by IL-8, which promotes the proliferation of stromal and epithelial cells (shown by green arrows). BPH cells secrete a lot of IL-12, IL-23p40, and IL-12p75, and they also activate alloantigen-specific CD4+ T cells, which help IFN-γ and IL-17 production. These two cytokines can also increase IL-8 output (shown by pink arrows). IFN-γ and IL-17 stimulate basal cells to secrete antigen-presenting and pro-inflammatory cytokines such as IL-6, IL-8, and CXCL10 (shown by blue arrows). IFN-γ may trigger the production of IL-15 by stromal cells (shown by yellow arrows).

A chronic inflammatory state may lead to tissue damage, activate the release of cytokines, increase the concentration of growth factors, and cause a local vicious cycle. Activated T cells release cytokines and interleukin related to cell injury, such as increased expression of IL-15 in stromal cells [53], increased expression of IL-17 in T cells [25], increased expression of interferon-γ in basal and stromal cells [54], and increased expression of IL-8 in epithelial cells. IL-8 is considered to play a significant role because it induces the expression of FGF-2, and FGF-2 has been proven to be an effective growth factor for stromal cells and epithelial cells [31] (Figure 1). This complex pro-inflammatory microenvironment is closely related to the excessive proliferation of BPH stroma, similar to the myofibroblast-based reactive matrix and extracellular matrix remodelling observed during wound repair in most inflammation-related fibrosis diseases [55]. These observations suggest that inflammation-induced prostate tissue injury represents a chronic process of wound healing. This process activates the overproliferation process that leads to BPH nodules. The specific roles of inflammation and proinflammatory cytokines in BPH are shown in Table 1.

### 4.1. IL-2 and BPH

IL-2 is a cytokine produced by the Th1 cell-mediated cellular immune response (Figure 1). In patients with BPH, the expression of interleukin-2 receptor (IL-2R) and IL-6 was higher in patients with prostatitis than in those without prostatitis [56]. In the early stage of BPH, IL-2 mRNA expression is tenfold higher than in normal prostate due to the predominance of Th1 immune response [1]. Moreover, a recent study showed that the expression level of IL-2 mRNA in normal prostate, noninvasive BPH specimens, and BPH nodules was lower than that in invasive prostate tissues [52]. However, the expression increased tenfold in BPH samples with normal histological infiltration and BPH samples without infiltration. It was also found that the tenfold increase in the expression of IL-2 mRNA in invasive BPH specimens with BPH histology was associated with a threefold increase in the expression of Interferon-γ (IFN-γ) mRNA [52]. In addition, gene expression profiling analysis in a rat model revealed that IL-2-related genes were relatively activated during the growth of BPH [57]. During the period of chronic inflammation, the secretion of IL-4 and IL-13 increased in the late stage of BPH, suggesting that the Th1 immune response will gradually shift to the Th2 immune response [52]. The immunological response that is prevalent in the development of BPH is dominated by distinct pro-inflammatory cytokines at different stages of development, as shown in the preceding study. The role of IL-2 in the early stages of immunological transformation will probably play a key role in aiding the subsequent immune transformation.

### 4.2. IL-8 and BPH

Human BPH cells express MHC Ⅱ molecules and costimulatory molecules (an important feature of APC), produce high levels of IL-12/IL-23p40 and IL-12p75, and activate alloantigen-specific CD4+ T cells to induce the secretion of IFN-γ and IL-17. These cytokines strongly promote the production of IL-8 by BPH cells [58] (Figure 1). IL-8, as an autocrine/paracrine growth factor of BPH cells, is a potential link between chronic inflammation and prostate enlargement [58,59,60]. It is considered to be the most important cytokine involved in the occurrence and development of BPH and can directly promote the proliferation of the epithelium and stroma [60,61]. The study has also shown that the expression levels of pro-inflammatory cytokines and chemokines IL-8, IL-6, IL-17 and CXCL10 in BPH tissues were different from those in normal prostate tissues. Among them, the transcriptional level of IL-8 is three to six times higher than that of IL-6, IL-17, CXCL12, CXCL10, and GM-CSF, indicating that IL-8 may be indispensable in the occurrence and development of BPH. Although there is no differential expression of IL-6 receptor transcripts, the transcriptional level of IL-8 and its receptors (R1 and R2) in BPH tissues is five to 25 times higher than that in normal prostate tissues. In addition, the levels of IL-8 receptor and R7 in BPH were more than five times higher than those in normal tissues [60].

These results indirectly suggest that the IL-8 axis plays a key role in the inflammatory environment of the prostate transitional zone, which contributes to the dilation and hypertrophy of the prostate gland [62]. Epithelial cells and prostatic epithelial progenitor cells from BPH tissue secrete high levels of IL-8 and initiate the inflammatory process by attracting immune cells, such as neutrophils and leukocytes, into the prostate. These immune cells in turn secrete IL-6 and IL-17 to form a pro-inflammatory microenvironment, resulting in a chronic inflammatory environment [62]. With the increase in the number of inflammatory cells and the continuous secretion of cytokines, the proliferation of epithelial and interstitial smooth muscle cells, glandular hyperplasia, and fibrosis are stimulated. BPH cells express both IL-8 homologous receptors CXCR1 and CXCR2. Recently, it has been found that the addition of a neutralizing anti-CXCR1 monoclonal antibody can significantly inhibit the autocrine/paracrine proliferation of BPH cells. In contrast, the inhibitory effect of an anti-CXCR2 monoclonal antibody on IL-8-induced proliferation of BPH cells was weak but not statistically significant, indicating that the autocrine/paracrine proliferation of IL-8 was mainly mediated by binding to the IL-8 receptor CXCR1 [58]. In particular, IL-8 is also considered to be a reliable biomarker of BPH [63]. Currently, research related to IL-8 is a hot topic, which may suggest that curing BPH from the inflammatory side is a feasible option.

### 4.3. IL-15 and BPH

IL-15 is a regulator of lymphocyte proliferation, migration, and homeostasis, and is an important cytokine that produces and maintains inflammatory infiltration in the prostate; it is expressed by normal prostate cells and is mainly limited to smooth muscle cells (SMCs) [64]. Handisurya et al. [53] found that IL-15 was overexpressed in BPH and could induce massive proliferation of BPH-T lymphocytes (Figure 1). The mRNA levels of IL-15 and its receptor (IL-15R) in the luminal secretory epithelium of BPH samples increased, as mentioned above, which may be due to the paracrine effect of IFN-γ, resulting in a twofold increase in IL-15 produced by stromal cells and lymphocyte activation and recruitment [53]. By further boosting IFN-γ production, IL-15’s involvement in sustaining inflammatory infiltration causes BPH.

### 4.4. IL-17 and BPH

IL-17 is a pro-inflammatory cytokine derived from lymphocytes. It is mainly expressed by activated prostate T lymphocytes and promotes the production of other pro-inflammatory cytokines [51,65]. Some studies have shown that compared with normal prostate tissue, the expression of IL-17 mRNA and protein in BPH is increased [51]. As mentioned above, BPH cells can activating alloantigen-specific CD4+ T cells to induce the secretion of IFN-γ and IL-17. IFN-γ and IL-17 can induce BPH cells to secrete IL-6, IL-8, stromal and epithelial growth factors, and strongly secrete CXCL10 [66,67]. Among them, the effect of IL-17 on the upregulation of IL-8 was stronger than that of IFN-γ. In contrast, CXCL10 was only induced by IFN-γ [58].

A recent study found that the proliferation of BPH cells was completely inhibited by neutralizing anti-IL-8 monoclonal antibodies, indicating that IL-8 produced by IFN-γ/IL-17 stimulation was involved in inducing the proliferation of BPH cells. In the presence of a CXCR1-specific neutralizing monoclonal antibody, the proliferation pathway of BPH cells induced by IFN-γ/IL-17 was blocked, but the IL-8-dependent loop was involved in the autocrine/paracrine proliferation of BPH cells. Arivazhagan et al. [68] found that IL-17 was significantly elevated in prostate tissue in BPH compared with controls, and it correlated with prostate size. Subsequently, Gao et al. [69] also showed that the expression of IL-17 was upregulated in the prostate tissue of BPH patients with inflammation. Another study examined the level of IL-17 in the serum of patients with BPH. It found that serum IL-17 levels in patients with BPH were significantly higher than those in controls [68]. By stimulating the synthesis of other pro-inflammatory molecules that contribute to the establishment of an inflammatory milieu, IL-17 modulates the adaptive immune response. It also significantly increases the release of IL-8, implying that IL-17 may play a crucial intermediary role in this complicated route.

### 4.5. IFN-γ and BPH

IFN is a glycoprotein that can regulate immune cells during the invasion of viruses, bacteria, parasites, and cancer cells. It is a part of the cytokine superfamily [70]. IFN-γ is a secretory protein. It belongs to the type II IFN family. It can be used as a regulatory factor to activate macrophages, or it can be activated by specific antigen presentation to induce apoptosis. In addition, IFN-γ can also induce the antitumor, antiviral, and immunomodulatory effects of type I IFNs, such as IFN-α, IFN-β, IFN-ω, IFN-κ, and IFN-ε [71,72,73]. The importance of IFN-γ in prostate cancer cannot be overstated. In an in vitro experiment, the results showed that IFN-γ and TNF-α could enhance the expression of IDO and IL-6 in prostate cancer cells at the gene and protein levels, and the data showed that the expression of IDO and IL-6 genes was higher in prostate cancer than in prostate hyperplasia, and that their expression was highly significantly correlated with the expression of IFN-γ and TNF-α genes [74].

In prostate cells, through the primary culture of prostate basal cells, it can be found that the basal cells stimulated by IFN-γ and IL-17 have the main characteristics of antigen-presenting cells and produce pro-inflammatory cytokines and chemokines, such as IL-6, IL-8, and CXCL10, to promote the inflammation of BPH to form a positive feedback loop [58]. Quantitative RNA analysis showed that the level of IFN-γ in BPH tissue was three times higher than that in normal prostate tissue. As a product of T cells, IFN-γ is overexpressed in BPH. In normal prostate tissue, stromal cells proliferate without the effect of IFN-γ. In contrast, stromal cells derived from BPH showed considerable proliferation promotion [67]. IFN-γ can cause stromal cells to produce IL-15, which is considered to be the most effective cytokine for maintaining T-cell infiltration in the prostate [53]. Therefore, the increased expression of IFN-γ may cause more T lymphocytes to be recruited to the BPH region, maintain the immune response, and develop into chronic inflammation (Figure 1). 

### 4.6. TGF-β and BPH

Transforming growth factor β (TGF-β) is an inflammatory cytokine secreted by T lymphocytes that plays an important role in regulating the proliferation and differentiation of the prostate stroma. It is the key factor of androgen regulating prostate growth [75]. The activation of androgen leads to an increase in the level of cytokines responsible for proliferation. As mentioned earlier, TGF-β1 can induce mesenchymal fibroblasts to differentiate into myofibroblasts, regulate the response of epithelial cells to the insulin-like growth factor (IGF-1)-mediated mesenchymal-epithelial cell axis [32,76], and participate in the process of prostate hyperplasia. Previous studies have shown that IL-17, IL-6, and TGF-β1 can provide a continuous positive loop and enhance the immune inflammatory process of BPH^25^ [77]. Descazeaud et al. [78] studied the expression of TGF-β receptor II (TGFBRII) protein in patients with BPH and found that there was a significant correlation between TGFBRII interstitial staining and prostate volume, and BPH inflammation was also related to TGFBRII staining. TGF-β plays a key function in the modulation of immunomodulatory responses once it is activated. Its involvement in cancer development has received much attention recently, and it plays a similar role in BPH development.

**Table 1:** Inflammation and proinflammatory cytokines in BPH.

Cytokine or Factor	Function in BPH	References
IL-2	Promotion of the cloning of BPH stromal cells	[1]
IL-8	Induction of autocrine/paracrine proliferation of BPH cells, a powerful growth factor in prostate stromal and epithelial cells	[1,59,60,61,62]
IL-15	Strong stimulation of the growth of BPH-T-lymphocytes	[53]
Il-17	Strong induction of IL-8 production by prostate epithelial and stromal cells	[1,58]
IFN-γ	Induction of IL-15 production by prostate stromal cells	[1,53]
TGF-β	Regulation of the proliferation and differentiation of the prostate stroma; Induction of epithelial–mesenchymal transition	[75,76]

BPH = benign prostatic hyperplasia; IFN-γ = Interferon γ; TGFβ = transforming growth factor β.

## 5. Therapeutic Targets for Inflammation

In the past few decades, several drugs targeting inflammation of the prostate have been proposed to control BPH and its symptoms. These drugs include phytotherapeutics, phosphodiesterase type 5 inhibitors (PDE5-Is), and non-steroidal anti-inflammatory drugs (NSAIDs). Among them, phytotherapy was the most widely reported.

At present, phytotherapeutics used include extracts or seeds of several different plants, such as *Saw palmetto*, *Cucurbita pepo* L., *Prunus africana*, *Urtica dioica* L., and *Secale cereale* L. [79,80] (Table S1). Numerous in vitro tests have shown that plant preparations can have anti-inflammatory, anti-androgenic, and proliferative effects [81]. Botanicals are considered a promising treatment for BPH, but given the methodological problems and heterogeneity of existing clinical trials, current guidelines do not recommend them as a standard of care for BPH or LUTS. 

Extracts of saw palmetto are the most commonly used; its anti-inflammatory activity has been most well-investigated in the past few decades. Studies have shown that the hexanic lipidosterolic extract of Serenoa repens (hexanic LSESr) could exert anti-inflammatory effects by inhibiting the production of cyclooxygenase 2 (COX-2) and 5-lipoxygenase (5-LOX) metabolites or by depressing the expression of inflammatory mediators such as monocyte chemotactic protein-1/chemokine (C-C) motif ligand 2 (MCP-1/CCL2) [82,83]. Navarrete et al. [84] demonstrated that the extract reduced the number of B lymphocytes and decreased interleukin-1β (IL-1β) and tumor necrosis factor-α (TNFα) levels in males with BPH. Some scholars have also found that hexanic LSESr may inhibit cell proliferation and induce cell apoptosis [85,86]. Vela-Navarrete and colleagues found enhanced bax/bcl-2 index expression and caspase-3 activity in prostate tissue from BPH patients treated with hexanic LSESr [87]. Therefore, HESr may play a role in regulating BPH immune cells and associated inflammatory infiltration [88]. Although a systematic review of the literature suggested that hexanic LSESr was effective in relieving urinary symptoms and improving urinary flow due to BPH compared with placebo [89], large randomized controlled trials are still needed to confirm the benefit of hexanic LSESr on BPH.

NSAIDs act on different parts of the arachidonic acid cascade and can inhibit the action of phospholipase A2, cyclooxygenase, and prostacyclin synthetase [90]. Previous studies have confirmed that traditional NSAIDs and COX-2 inhibitors could reduce the clinical symptoms of patients with LUTS [91]. A pooled analysis of 183 men from three randomized placebo-controlled trials (lasting 4–24 weeks) showed that NSAIDs improved urinary symptom scores and peak urinary flow rate. However, their long-term effectiveness and safety remains unclear [92]. 

PDE5-Is have been considered as an effective treatment for patients with BPH. Numerous studies have shown that they can alleviate the clinical symptoms of patients by downregulating prostate inflammation [93]. Vignozzi et al. [94] reported that tadalafil or vardenafil could inhibit IL-8 secretion induced by either TNF-α or metabolic factors. In addition, a recent study found that the cyclic guanosine monophosphate/protein kinase G (cGMP/PKG) signal pathway activated by PDE5-I can suppress the infiltration of CD8 T-cell and the expression of CCL5 and cyclin D1, thus inhibiting the proliferation of BPH epithelial cells [95]. 

Recently, Vickman and colleagues reported that TNF may be a potential therapeutic target for suppressing BPH in autoimmune disease (AI) [96]. They evaluated 112,152 male patients and found that the prevalence of BPH in patients diagnosed with AI disease increased significantly. Further analysis revealed that the use of TNF antagonists significantly reduced the incidence of BPH. Moreover, single-cell RNA-seq and in vitro assays showed that macrophage-derived TNF stimulated the proliferation of BPH-derived fibroblasts. TNF blockade could significantly reduce epithelial hyperplasia, NFκB activation, and macrophage-mediated inflammation in prostate tissues [96]. Together, these results suggest that TNF antagonists can be used to reduce inflammation and inhibit BPH. However, further studies are needed to confirm the efficacy and safety of TNF antagonists.

## 6. Conclusions

To date, the pathogenesis of BPH is not fully understood. It is a complex process. Inflammation appears to play a major part in this process. A large number of inflammatory cells and inflammatory factors constitute the inflammatory microenvironment in the prostate tissue, which causes a great amount of T and B lymphocytes and macrophages to invade the prostate tissue while also stimulating inflammatory cells to secrete a high proportion of inflammatory cytokines. In prostatic hyperplasia with histological inflammation, the detection of relevant cytokines is an important guide to further understanding the pathogenesis of prostatic hyperplasia. In contrast, histologic inflammation and clinical studies can stratify the risk associated with lower urinary tract symptoms in patients and provide new therapeutic strategies for the treatment of BPH with histologic inflammation.

## Data Availability

Not applicable.

## Supplementary Materials

The following supporting information can be downloaded at: https://www.mdpi.com/article/10.3390/jcm12051821/s1, Table S1. Phytotherapeutics for the treatment of benign prostatic hyperplasia and their mechanism of action. References [97,98,99,100,101,102,103,104,105,106,107,108,109,110] are cited in the Supplementary materials.
