# Supplementary material for: A Novel Insight into the Immune-Related Interaction of Inflammatory Cytokines in Benign Prostatic Hyperplasia
Source: J Clin Med. 2023 Feb 24;12(5):1821. doi: 10.3390/jcm12051821 (PMC10003138; doi:10.3390/jcm12051821)
Supplement: Supplementary file 1 [file jcm-12-01821-s001.zip › jcm-2084895-supplementary.pdf]

Table S1. Phytotherapeutics for the treatment of benign prostatic hyperplasia and their mechanism of action.

**Plant Species**	**Postulated mechanism of action**	**References**
Serenoa repens (saw palmetto)	• 5α-reductase inhibition<br>• decrease of DHT level (Inhibiting its binding to androgen receptors)<br>• inhibits DHT and some testosterone metabolites formation<br>• anti-inflammatory activity<br>• anti-proliferative activity<br>• spasmolytic effects<br>• α1-adrenoceptors antagonism	[1,2]
Cucurbita pepo	• Decrease of DHT level (Inhibiting its binding to androgen receptors)<br>• 5α-reductase inhibition<br>• Inhibition of testosterone-induced hypertrophy	[3,4]
Prunus africana	• Anti-inflammatory activity<br>• anti-proliferative activity<br>• pro-apoptotic activity<br>• Inhibition of estrogen receptors, pro-gesterone and androgen receptors.	[5–7]
Urtica dioica	• Anti-proliferative activity<br>• anti-inflammatory activity<br>• Inhibits TNF activity<br>• Inhibits the connecting of SGBG<br>• Stimulates T-lymphocytes activity and the complement activation	[8–11]
Secale cereale	• Anti-inflammatory activity-decreased IL-6 and TNF-α cytokines levels<br>• pro-apoptotic activity<br>• α-adrenergic receptors block<br>• spasmolytic activity<br>• 5α-reductase inhibition<br>• Decreased PSA expression and androgen receptor	[12–14]

DHT = dihydrotestosterone; TNF = tumor necrosis factor; SGBG = sex hormone binding globulin; IL = interleukin; PSA = prostate-specific antigen

References

1. Gravas, S.; Samarinas, M.; Zacharouli, K.; Karatzas, A.; Tzortzis, V.; Koukoulis, G.; Melekos, M. The effect of hexanic extract of Serenoa repens on prostatic inflammation: results from a randomized biopsy study. World J Urol 2019, 37, 539-544, doi: 10.1007/s00345-018-2409-1.

2. Ishii, I.; Wada, T.; Takara, T. Effects of saw palmetto fruit extract intake on improving urination issues in Japanese men: A randomized, double-blind, parallel-group, placebo-controlled study. Food Sci Nutr 2020, 8, 4017-4026, doi: 10.1002/fsn3.1654.

3. Tsa,i Y.S.; Tong, Y.C.; Cheng, J.T.; Lee, C.H.; Yang, FS.; Lee, H.Y. Pumpkin seed oil and phytosterol-F can block testosterone/prazosin-induced prostate growth in rats. Urol Int 2006, 77, 269-74, doi: 10.1159/000094821.

4. Gossell-Williams, M.; Davis, A.; O'Connor, N. Inhibition of testosterone-induced hyperplasia of the prostate of sprague-dawley rats by pumpkin seed oil. J Med Food 2006, 9, 284-6, doi: 10.1089/jmf.2006.9.284.
5. Paubert-Braquet, M.; Cave, A.; Hocquemiller, R.; Delacroix, D.; Dupont, C.; Hedef, N.; Borgeat, P. Effect of Pygeum africanum extract on A23187-stimulated production of lipoxygenase metabolites from human polymorphonuclear cells. J Lipid Mediat Cell Signal, 1994, 9, 285-290.
6. Quiles, M.T.; Arbós, M.A.; Fraga, A.; de Torres, I.M.; Reventós, J.; Morote, J. Antiproliferative and apoptotic effects of the herbal agent Pygeum africanum on cultured prostate stromal cells from patients with benign prostatic hyperplasia (BPH). Prostate 2010, 70, 1044-1053, doi: 10.1002/pros.21138.
7. Papaioannou, M.; Schleich, S.; Prade, I.; Degen, S.; Roell, D.; Schubert, U.; Tanner, T.; Claessens, F.; Matusch, R.; Baniahmad, A. The natural compound atraric acid is an antagonist of the human androgen receptor inhibiting cellular invasiveness and prostate cancer cell growth. J Cell Mol Med 2009, 13, 2210-2223, doi: 10.1111/j.1582-4934.2008.00426.x.
8. Jonler, M.; Riehmann, M.; Brinkmann, R.; Bruskewitz, R.C. Benign prostatic hyperplasia. Endocrinol Metab Clin North Am 1994, 23, 795-807.
9. Durak, I.; Biri, H.; Devrim, E.; Sözen, S.; Avci, A. Aqueous extract of Urtica dioica makes significant inhibition on adenosine deaminase activity in prostate tissue from patients with prostate cancer. Cancer Biol Ther 2004, 3, 855-857, doi: 10.4161/cbt.3.9.1038.
10. Konrad, L.; Müller, H.H.; Lenz, C.; Laubinger, H.; Aumüller, G.; Lichius, J.J. Antiproliferative effect on human prostate cancer cells by a stinging nettle root (Urtica dioica) extract. Planta Med 2000, 66, 44-47, doi: 10.1055/s-2000-11117.
11. Chung, L.W.; Matsuura, J.; Rocco, A.K.; Thompson, T.C.; Miller, G.J.; Runner, M.N. Tissue interactions and prostatic growth: a new mouse model for prostatic hyperplasia. Ann N Y Acad Sci, 1984, 438, 394-404, doi: 10.1111/j.1749-6632.1984.tb38301.x.
12. Kimura, M.; Kimura, I.; Nakase, K.; Sonobe, T.; Mori, N. Micturition activity of pollen extract: contractile effects on bladder and inhibitory effects on urethral smooth muscle of mouse and pig. Planta Med 1986, 2, 148-51, doi: 10.1055/s-2007-969102.
13. Yasumoto, R.; Kawanishi, H.; Tsujino, T.; Tsujita, M.; Nishisaka, N.; Horii, A.; Kishimoto, T. Clinical evaluation of long-term treatment using cernitin pollen extract in patients with benign prostatic hyperplasia. Clin Ther 1995, 17, 82-87, doi: 10.1016/0149-2918(95)80009-3.
14. Rugendorff, E.W.; Weidner, W.; Ebeling, L.; Buck, A.C. Results of treatment with pollen extract (Cernilton N) in chronic prostatitis and prostatodynia. Br J Urol 1993, 71, 433-8, doi: 10.1111/j.1464-410x.1993.tb15988.x.
